# Collateral Damage: Reuse in the Arts and the New Role of Quotation Provisions in Countries with Free Use Provisions After the ECJ’s *Pelham*, *Funke Medien* and *Spiegel Online* Judgments

**DOI:** 10.1007/s40319-021-01084-4

**Published:** 2021-07-05

**Authors:** Alan Hui, Frédéric Döhl

**Affiliations:** 1 RITMO Centre and Department of Musicology, The University of Oslo, Oslo, Norway; 2 Free University, Berlin, Germany; 3 German National Library, Leipzig, Germany

**Keywords:** Copyright, Quotation, Reuse, Free use, EU

## Abstract

This article examines the impact of the European Court of Justice’s *Pelham* decision (C-476/17) on reuse, including appropriation art, borrowing and plagiarism in the arts, especially in music. Insofar, the focus lies on countries that have operated before with broad free use provisions. Specifically, we consider the extent to which EU law permits quotation provisions to fill the gap left by free use limitations, which have been curtailed by the *Pelham* decision. As we explain, *Pelham* creates a more restrictive approach to certain unlicensed use of copyright materials in new works of artistic expression, including music appropriation. We present our research in four sections. First, we compare existing national free use and quotation provisions in four states subject to EU law regarding their respective wiggle room for unlicensed yet lawful reuse in the arts. Second, we explore how the ECJ’s interpretation of the InfoSoc quotation exception, particularly in the *Pelham*, *Funke Medien* and *Spiegel Online* judgments, minimises the leeway for reuse in the arts provided by these national quotation provisions, in comparison to free use provisions. (Information Society Directive, 2001/29/EC.) Third, we address possible objections to our position and explain why we believe the consequences of the ECJ decisions cannot be bypassed. (Copyright in the Digital Single Market Directive, (EU) 2019/790.) Finally, in the conclusion, we explore the consequences, including the need for legislative reform.

## Introduction

This article deals with the field of reuse in the arts, especially in music.^1^ It addresses the present state of copyright and related rights (especially the rights of performers and phonogram producers) regulation of reuse in the single market of the European Union (EU). The heart of our analysis is the lawfulness of unlicensed yet free use of protected third-party materials (works, performances, and media) in new works of artistic expression.

In this article, we give special attention to the recent decisions of the European Court of Justice (ECJ) and its impact on reuse and music quotation.^2^ This trio of ECJ copyright decisions in 2019 locks EU and EEA states into the Information Society Directive (InfoSoc Directive) framework of specific exceptions and limitations.^3^ We focus specifically on the remaining flexibility in quotation exceptions dealt with in the ECJ’s *Pelham*, *Funke Medien*, and *Spiegel Online* judgments after the ECJ’s *Pelham* decision reduced the ability for reuse in the arts to rely on national free use provisions (also known as “independent work” or “new work” limitations; in this article, we use “free use” as an umbrella term). Overall, such free use provisions fell victim to a conflict about something that is only related in specific cases to the question of unlicensed yet lawful reuse in the arts: the exclusive reproduction right of phonogram producers. Yet, in our view, the ECJ’s categorial opposition to national free use provisions as standing outside InfoSoc’s legal framework in *Pelham* affects all areas of reuse in the arts, not just those where the reproduction right of phonogram producers is involved – creating in our estimation significant collateral damage for the arts. This leads to the significant challenge of how to contain this collateral damage and our main question in this article: To which extent can quotation provisions assume the role of free use provisions in cases of reuse in the arts?

Due to this, we are interested in the impact of the recent judgments of the ECJ on countries which operated with broad free use limitations that were essential for most cases of reuse in the field of arts. We draw upon, as case studies, laws from four nations subject to EU law as member states of the EU (Germany, Netherlands, Sweden) and the EEA (Norway) that have had both free use and quotation provisions.^4^ We include the Nordic states of Sweden and Norway, noting that while they retain historical similarities in their copyright legislation, interpretation of these statutes may vary going forward. This is partly because Norway is a member of the EEA, unlike Sweden which is an EU state, and therefore subject to the EEA principle of homogeneity, which creates a special relationship between EU case law and EEA national case law.^5^ In addition, as we will discuss in the following section, the statutes vary in some ways across the two jurisdictions.

In our view, the recent trio of ECJ copyright decisions holds severe consequences for these countries. Together, they clarify that there is no room for either national exceptions not listed in the InfoSoc Directive or national courts to interpret exceptions permitted in EU law differently from the ECJ. By ruling in *Pelham* that a member state “cannot, in its national law, lay down an exception or limitation other than those provided for in Article 5 of Directive 2001/29”, the ECJ closed the door on the more general free use exceptions that were in place in these four jurisdictions.^6^ At the same time, the ECJ’s *Pelham*, *Spiegel Online* and *Funke Medien* decisions collectively restrict quotation with new requirements that minimise the wiggle room for unlicensed yet lawful reuse in the arts in existing national legal regimes (see Sects. 2 and 3). As we will discuss, our view remains true as long as one

- does not interpret the ECJ’s position in a way that national free use provisions could be retained by way of national discretion regarding the requirements of the InfoSoc Directive (see Sect. 4.1);^7^
- accepts that the ECJ’s recent judgments in *Pelham*, *Spiegel Online* and *Funke Medien* block at least for the time being any chance of (1) narrowing reproduction rights or (2) amending listed exceptions and limitations by way of court decisions with regard to EU fundamental rights, especially the freedom of the arts (Art. 13 CFR, see Sect. 4.2);^8^
- does not interpret the still untested InfoSoc pastiche exception, which has become mandatory with the DSM Directive, to assume the function of the national free use provisions (see Sect. 4.3);^9^
- does not interpret the quotation provision of the InfoSoc Directive in much broader terms than applied by the ECJ so that it at least strives to and comes close to superseding the former free use provisions in all but name (see Sect. 4.4).^10^

For the countries which, in our understanding, basically lost their free use provisions, the trio of ECJ decisions and the DSM Directive create an urgency for most forms of reuse in the arts to clarify the framework of the EU quotation exception.^11^ Its national equivalents offer the best – and in our view unfortunately, in fact, only – chance to save significant room for cases of unlicensed yet lawful reuse in the arts. For this reason, our article examines, first, whether the respective national quotation provisions are consistent with the InfoSoc Directive and, second, what requirements are imposed.^12^ Especially in the case of countries that have had free use limitations on copyright and related rights, which are now illegal following recent ECJ judgments, these legal developments constitute in our view a substantial change of system and a real gamechanger.^13^ This is why we deem it useful to check realistically how broadly the respective quotation provisions can be interpreted in the face of the present national and EU requirements and how great a gap remains compared to the respective former free use provisions.

Although quotations exist in a variety of art and expressive forms, this article will focus on music examples. Musical forms of different cultures, times and places present a smorgasbord of rearrangement, appropriation and quotation which blurs the boundaries between quotations and independent works. Motets, themes and variations, jazz, dub, and hip-hop are some of these forms. Through the public web, online communities and content platforms, further forms have emerged, including mashups, anime music videos, megamixes and songification of news. Each brings its own aesthetic and social conventions of taking over third-party material. Free use limitations effectively accommodate such cultural diversity. But does the legal concept of quotation also make such accommodations? And if not, what are the consequences?

We present our research in four sections. First, we compare in Sect. 2 existing national free use and quotation provisions in four states subject to EU law regarding their respective wiggle room for unlicensed yet lawful reuse in the arts. Section 3 explores how the ECJ’s interpretation of the InfoSoc quotation exception minimises the leeway for reuse in the arts provided by these national quotation provisions, in comparison to free use provisions.^14^ Section 4 addresses possible objections against our position and explains why we believe the consequences of the ECJ decisions cannot be bypassed.^15^ Finally, in the conclusion we explore consequences, including the need for legislative reform.

## Gaps Between Free Use and Quotation in National Laws

National copyright provisions permitting “free use” of protected third-party materials to create new works in the arts have been important for many reasons. Creativity in the arts almost always draws on conventions or common building blocks expressed in existing works. There is no art without engaging with what has come before. Connections and similarities to other works are often a key factor, even in cases without reuse or appropriation. Free use permitted some adapting and transforming of third-party works, which has become a widespread social phenomenon in digital contexts, including user-generated content (beyond the realm of professional artists) and social media communication. Free use has also been important as there has been no system of compulsory licensing for adaptations in the single market (as is the case for covers of musical works) and establishing one would face notable barriers such as moral rights. Likewise, ad hoc licensing is very difficult as the German Constitutional Court recognised during the *Pelham* case in 2016 with regard to sound sampling.^16^ Furthermore, European copyright law on EU and member state levels finds infringement not just in deliberate reuse but also in accidental and even subconscious acts of appropriation. This arguably creates an almost absolute scope of protection in copyright, crowding out both the possibility of non-infringing, parallel, independent creation and the “requirement of availability”.^17^ By contrast, free use provisions have had the essential function of balancing the scope of protection and therefore the freedoms of expression and the arts against the right to property.^18^

As the ECJ ruled in *Pelham*, nations in the single market cannot introduce new or retain existing free use limitations.^19^ Against the intentions of the German Federal Court of Justice to restrict its referral question to free use and phonogram rights, the ECJ’s decision expanded its answer to all InfoSoc rights and confirmed a strict interpretation concerning the scope of protection granted with the InfoSoc Directive.^20^ It explained that EU member states could implement only those exceptions listed in the InfoSoc Directive, such as quotations for “purposes such as criticism or review” and for “caricature, parody and pastiche” but not for the unlicensed yet free use of protected third-party materials.^21^ The German Federal Court of Justice’s latest *Pelham* judgment confirms the ECJ *Pelham* judgment, limiting free use from 22 December 2002 in relation to all InfoSoc rights.^22^ This follows the Berlin Court of Appeal’s ruling in late 2019 against internationally renowned appropriation artist Martin Eder, in which his appropriation of an artwork by a UK artist was not found to be free use (and not parody or quotation).^23^ German legal scholars hold a similar view to the German courts.^24^ Likewise, the German government also expressed this view while proposing the legislative repeal of the Sec. 24(1) free use provision, not limited to phonogram rights, explicitly naming the ECJ’s judgment as the reason for repeal.^25^ In light of these ECJ and German court decisions, unless EU legislators introduce free use into EU law, which would be legally possible yet seems unlikely given this did not occur in the DSM Directive, existing free use limitations are ineffective to the extent they exceed the scope of EU limitations and exceptions.

There is now an urgent need – in light of recent cases including the 2019 trio of *Pelham*, *Spiegel Online* and *Funke Medien* – to identify whether some forms of free use would also qualify as quotation under national laws and under EU law.^26^ In this section, we explore the scope of national quotation exceptions, particularly how much they may retain, enact, apply, or even extend the wiggle room for unlicensed yet lawful reuse of protected third-party materials in the arts. This lays the ground for considering gaps between national quotation provisions and InfoSoc quotation requirements in the following section.

We focus on quotation provisions because we see them as a key future battleground to balance the interests involved.^27^ In doing so, we align with Bently and Aplin who look to quotation provisions to salvage flexibility in copyright.^28^ We also align with Senftleben who looks to a broad court interpretation of quotation, cognizant of the need to balance fundamental rights, as a possible path to salvage the ECJ’s creation of a “flexibility grave”.^29^ However, we are less optimistic about the breadth of uses permitted by quotation provisions. As we explore in this section, the domestic quotation provisions come with a rather extended catalogue of requirements. And as we explain in the next section, InfoSoc requirements for quotation create some further challenges for national quotation provisions. We are also less optimistic about the steps necessary to proceed within the international legal framework described by Aplin and Bently to develop the quotation provision into a free use equivalent (see Sect. 4.4).^30^

Although we focus on quotation, we recognise there are at least four other carve-outs from InfoSoc rights which also remain on the table for reuse.^31^ Where there is no relevant licence, these carve-outs may also permit artistic expression that is identified as a conscious or unconscious, deliberate or accidental reuse of at least parts of a third-party work, performance or phonogram. As we explain below, in our view, however, these four carve-outs have more limited scope than quotation consistent with InfoSoc and ECJ requirements.

Firstly, a public domain carve-out permits use of a work, performance, recording, or part thereof in a new work; however, reuse in general and music appropriation in particular often draw on contemporary culture and media which are still protected.^32^ This is especially true for sound sampling. Despite being invented in the 1870s, sound recordings have achieved a sound quality since the 1950s that elevates them to contemporary sounding entities (instead of merely historical sound artifacts). Consequently, the market in more recent, protected recordings dwarfs the public domain of unprotected sound recordings with suitable sound quality for music appropriation artists to work with.^33^

Secondly, while using a part without originality is unrestricted in theory, proving lack of originality is difficult in practice. A part of the work or performance (but not recording) identified in the new work may not meet the necessary threshold of originality, being too insignificant and aesthetically basic; think of musical elements like general forms, chords, scales, rhythms, genres, styles and instrumentation. In such cases, the part is free to use for everyone and not restricted by the InfoSoc Directive.^34^ It protects a kind of “requirement of availability” to safeguard the possibility of future music-making.^35^ The difficulty, however, is whether the threshold of originality protects even some common and socially relevant artistic elements.

Thirdly, the *Pelham* unrecognisability test permits an uncertain range of uses. Under the test, using part of a phonogram modified in a way that makes it unrecognisable is not restricted by Art. 2(c) reproduction rights.^36^ It is still unclear whether this test will also apply to works and performances (as opposed to being restricted only to phonograms), though we believe this is likely.^37^ It is also unclear what constitutes unrecognisability, and whose ability to recognise matters (e.g. lay or qualified persons; humans or algorithms).^38^ In any case, we expect a modification will only be considered unrecognisable where the source of the used part is not attributed but implied only by external factors such as previous licensing requests or artist statements acknowledging the act of appropriation (as in the infamous *Blurred Lines* case).^39^

Finally, national exceptions for caricature, parody, or pastiche seem to permit only limited uses of a work, performance, recording or part thereof in a new work.^40^ As these are also autonomous concepts of EU law, they need to be defined by the ECJ. In the case of parody, *Deckmyn* limits a permitted use to specific, privileged artistic purposes that are humorous or mocking expressions that evoke an existing work while being noticeably different from parodied material.^41^ This may be difficult to establish, for example, in cases of sound adaptation without lyrics or visual accompaniment which need extensive musical knowledge typically beyond a lay listener’s ability.^42^ It is also difficult to “safeguard” the “fair balance” between the competing fundamental rights, as the InfoSoc Directive states, in cases of caricature and parody.^43^ Separately, what the category of pastiche stands for is still an open question in EU legal terms.^44^ Pastiche is an underdeveloped legal concept compared to quotation and parody, widely disputed in its meaning, shape, and content, and still undefined at an EU level by the ECJ.^45^ The pastiche exception could be developed into a broad exception, as currently proposed in Germany, but we are sceptical of this possibility, taking into account the aesthetic as well as legal origins of the term (see Sect. 4.3).^46^

Taking into account these characteristics and far-reaching restrictions of the aforementioned alternative four carve-outs, we expect that the discussion regarding scenarios for unlicensed yet lawful reuse in the arts will refocus on the national quotation provisions. We now consider in turn free use provisions, quotation provisions, and the gaps between free use and quotation provisions in the four countries, as well as between national and EU quotation law.

### Free Use: The National Concepts in Comparison

Free use is a purely national concept. Neither EU legal texts (such as the InfoSoc Directive) nor international copyright conventions or treaties (such as the Berne Convention) recognise free use as a copyright exception or limitation. Though the exact statutory language varies, at the core of each national free use provision is that use of material protected by copyright or related rights does not infringe when it yields a standalone work. For example, the German provision requires that the use yield an “independent” work, while the Dutch provision requires a “new” and “original” work.^47^ Likewise, the Norwegian and Swedish free use provisions require a “new” and “independent” (literally “self-standing”) work.^48^

Over several decades, national courts have elaborated on uses that fall under the umbrella of free use and how they carve out of copyright and related rights. In the context of free use, different explanations exist as to which similarities with the used work are infringing. For example, the German free use provision permits uses creating distance between the old and new materials. In general, courts expected the aesthetic identity and originality of the work or part used to fade into the background in an independent work.^49^ The original was allowed to still shine through, meaning the original is allowed to be experienced but not at the forefront of the new musical context. So, the free use provision addresses a unique yet artistically important area between adaptation in a narrow sense like translation or arrangement (needing a licence) and mere inspiration by a third-party work (needing no licence). Due to this, courts did not expect the used work to be unrecognisable in the independent work (as the ECJ has now required in *Pelham*).

Such free uses are distinguishable from copyright-infringing adaptations. In *Aukruststiftelsen v. Caprino Filmcenter*, for instance, the Norwegian Supreme Court found that a rollercoaster carriage built on the basis of drawings by Norwegian artist Kjell Aukrust did not infringe the copyright in a car which was an adaptation from the same drawings.^50^ The rollercoaster carriage had been made with the permission of the drawing’s artist but not the rightholder of the car. The Norwegian Supreme Court explained that an adapter must make sufficiently original changes through independent intellectual creativity to give the adaptation the status of a new work which stands on its own feet; the copyright protection in this new work excludes anything protected by the copyright in the original work.^51^

In general, the necessary independence is achieved by fading the old work into the background on a sensory level, for example by transforming a sound or making it otherwise less audible by way of its new context.^52^ Likewise, in a decision concerning imitations of wooden figurines, the Dutch Supreme Court established that two works looking alike by virtue of following the same method or style, on its own, is not sufficient to find infringement.^53^ In the *Una Voce Particolare* decision, the Dutch Supreme Court ruled that the overall impression of the two works must be taken into account, to the extent that impression arises from similar copyrighted elements.^54^

However, in cases where fading was not achieved (or intended), independence could be achieved through humorous and/or critical modifications like in parodies which create an “inner distance” between meanings of the old and new works. On many occasions, the national high courts have considered free use to include humorous-critical uses such as parodies and caricatures. The Swedish Supreme Court, for example, has established in successive cases that the new and independent provision can permit parodies and caricatures.^55^ In the *Sveriges Flagga* decision, the Swedish Supreme Court found that a song which reused the melody and some lyrics from an earlier song (which was set to the poem of the same name “The Flag of Sweden”) was not a parody and therefore not a free use. Whereas the earlier song was a tribute to the Swedish flag, the later song included different lyrics about the Vietnam War and advocated the burning of the American flag. The Court recognised that a parody would be permitted by the new and independent work provision but considered the later song did not target or ridicule the earlier song, and therefore was not a parody of the earlier song. Likewise, in the *Bettina Z* case, the German Federal Court of Justice recognised the functional equivalence of the German free use limitation to the InfoSoc parody exception in cases of parody.^56^ In *JL v. MA*, the Swedish Supreme Court found that an oil painting was a caricature and therefore free use.^57^ In the 1990s, media outlets featured a photograph of a person acquitted of murder. Later, an artist created an oil painting called Swedish Scapegoats, featuring a goat, the acquitted man and the photograph, without the photographer’s permission.^58^ The Swedish Supreme Court considered the painting to be a caricature, with a strongly contrasting meaning to the photograph thanks to its dull colours, harsh landscape and inclusion of a goat. The Court considered that the stronger the original work, the more difficult it may be to create a new work from that original work.^59^ In *GB v. Sveriges Radio*, the Court considered a parody or caricature could be a free use, but ruled that a humorous compilation of several literary works and films into a radio programme was instead a collage.^60^ The Court cited Olsson’s treatise on Swedish copyright, opining that a new and independent work must not be an adaptation, must retain the inner form of the original, and is mainly about whether the original has inspired the later author.^61^

Germany’s free use provision, however, possesses a unique feature in how it applies in the context of music. It has typically been limited to use of material of very limited originality and, since its inception in 1902, the statutory provision has specifically excluded the use of recognisable melodies from musical works, attracting significant criticism.^62^ Legal uncertainty arose from the most notable court cases dealing with deliberate or accidental reuse of parts at the threshold of originality, using very short parts of music. These factors have prevented a coherent free use concept in the field of music compared to other fields of art like theatre, writing, and visuals. However, as the exclusion of melodies from free use would be repealed with the proposed German implementation of the DSM Directive, it is not necessary to assess its impact. The exclusion of melodies from the German quotation provisions remains relevant, as we discuss below.

### Quotation: The National Concepts in Comparison

In contrast to the free use provisions, the respective quotation provisions have roots in various EU and international agreements. The EU InfoSoc Directive provides an exception for

quotations for purposes such as criticism or review, provided that they relate to a work or other subject-matter which has already been lawfully made available to the public, that, unless this turns out to be impossible, the source, including the author’s name, is indicated, and that their use is in accordance with fair practice, and to the extent required by the specific purpose.^63^

Under the Berne Convention, to which all EU and EEA states are signatories, it is permissible to quote from a work “which has already been lawfully made available to the public, provided that their making is compatible with fair practice, and their extent does not exceed that justified by the purpose, including quotations from newspaper articles and periodicals in the form of press summaries”.^64^ By being signatories of the WIPO Copyright Treaty, all EU states also affirm their compliance with certain parts of the Berne Convention, including the Art. 10(1) quotation provision.^65^ Quotation is at times even referred to as a right, as opposed to an exception or limitation, in and beyond national contexts. The Dutch Supreme Court has recognised the quotation provision as a right, referring to it as “*citaatrecht*”.^66^ Swedish and Norwegian rightholders sometimes refer to this provision as a quotation right.^67^

Quotation provisions in national jurisdictions which also recognise free use, however, vary in several important ways which we highlight here. This potentially means that these countries are impacted differently by the *Pelham* decision and its opposition to free use provisions. The table below provides an illustrative overview of some differences between quotation provisions in EU and EEA states which have also had a free use provision; these features are discussed in depth following Table 1.

**Table 1:** Similarities between selected quotation provisions

Characteristic of quotation provision	Germany	Netherlands	Norway	Sweden
Purposes limited to “such as criticism or review” (InfoSoc)	No	No	No	No
In accordance with good/fair practice (Berne)	No	No	Yes	Yes
To the extent required for the specific purpose (Berne, InfoSoc)	Yes	Yes	Yes	Yes
Extends to use of works and other subject matter (InfoSoc)	Yes	Yes	Yes	Yes
Limited to use of published works (Berne) (Typically, a work made available to the public is considered published in the national laws we consider. For examples, *see* Dutch Copyright Act 2018, Art. 47(3); and Swedish Copyright Act 1960:729, Sec. 8.)	Yes	Yes	Yes	Yes
Limited to use of works lawfully made available to the public (InfoSoc)	Yes	Yes	Yes	Yes
Requires attribution unless unreasonable (Berne)	No	Yes	Yes	Yes
Requires attribution unless impossible (InfoSoc)	Yes	No	No	No
Subject to moral right of integrity (prohibition of alteration) unless impossible or unreasonable (Berne)	Yes	Yes	Yes	Yes
Quotation as a right (Berne)	Yes	Yes	Yes	Yes
Free of charge	Yes	Yes	Yes	Yes

#### Purposes

First and foremost, different jurisdictions have different requirements regarding the purposes for quotation. The Dutch provision only permits quotation that meets one or more of the listed purposes, including to quote “in an announcement, review, polemic or scientific treatise or piece with a comparable purpose”, “in the form press surveys of articles appearing in a daily or weekly newspaper or other periodical”, and “quotations in a language other than the original”.^68^ The German provision has no catalogue of privileged purposes, though the commercial exploitation of the quoted work must not be harmed, which affects those quotations with a commercial purpose or effect.^69^ The Swedish and Norwegian provisions do not limit purposes, though like the Dutch and German provision, they provide an exception for both citation and quotation; this is *prima facie* broader than an English provision for quotation only.^70^

#### The Extent Required by the Purpose

Some national quotation provisions also link how much can be quoted to the purpose itself. The Norwegian, Swedish and Dutch provisions explicitly link the extent of permissible quoting to the purpose.^71^ The Dutch provision has a more specific requirement that “the number and size of the quoted parts are justified by the purpose to be achieved”.^72^ The Norwegian Supreme Court has also articulated how the extent requirement can apply in a Norwegian context. In *NRK v. Mauseth*, the Norwegian Supreme Court found the broadcast of excerpts from a film infringed the performer’s rights of actress Gørild Mauseth and was not lawful quotation. A television review of a parody film showed excerpts of a sex scene from the parodied film, including two seconds of nudity featuring Mauseth. The Supreme Court upheld a lower court ruling that the excerpt exceeded the extent required by the purpose of the quotation and therefore infringed the performer’s right.^73^ In the Supreme Court’s view, the purpose justified showing part of the sex scene but not the two seconds of nudity. Although the court found there was not a permissible quotation, it recognised a link between quotation and freedom of speech.^74^

#### In Accordance with Certain Practice or Custom

Related to the purpose of the quotation is the requirement that quotation be conducted in accordance with certain practice. Whereas the Norwegian and Swedish statutes permit quotation “in accordance with good practice”, the Dutch statute permits quotation “in accordance with what social custom regards as reasonably acceptable”.^75^ The German statute is silent on good or fair practice, though current interpretation requires that the quoted work must become a substantial part of the quoting work which must also form a standalone work if the quoted material is taken away (presenting a significant problem for everything from collage to mashup). The quotation must be more than ornamentation and generate additional musical meaning within the context of the new work.

#### Extends to the Use of Works and Other Subject Matter

National quotation provisions also permit quotation of a range of the subject matter of copyright and related rights, though the exact range varies. The Swedish statute extends the quotation provision for works to also carve out of related rights in Chapter 5. Likewise, the Norwegian statute extends the quotation provision for works to also carve out of related rights in Chapter 2. Regarding most related rights, the German Act includes in the respective provisions an explicit extension of the quotation exception to the respective related right. The Dutch quotation provision covers a range of materials; although the statute mentions only “the copyright in a literary, scientific or artistic work”, the official government document accompanying the amendment explains the provision can permit “sound quotes” (*klank-citaat*), amongst quotes of other materials.^76^

#### Quoted Subject Matter Lawfully Made Available to the Public

Generally, national quotation provisions include some requirement that the quoted subject matter is publicly available. The German, Norwegian and Swedish provisions require only that the quoted work is published, making no distinction between lawful and unlawful publication.^77^ The Dutch provision requires that the quoted subject matter is *lawfully* made public.^78^

#### Attribution and Alteration

Regardless of the subject matter used, national quotation provisions typically require attribution. Different jurisdictions specify different circumstances where attribution is required. Under the German provision, the source must be attributed when communicating quotation of a work to the public.^79^ When reproducing or distributing a copy of a work, the source must be attributed except if the source is not known to the person making the quotation.^80^ The Norwegian provision requires attribution where practically possible.^81^ The Dutch provision requires indication of the source where reasonably possible.^82^ The Swedish provision requires attribution of the source “to the extent and in the manner required by good practice”, but does not explicitly contain the requirement to cases where attribution is possible.^83^

In addition to attribution, national quotation provisions are also subject to alteration restrictions. The Norwegian and Swedish statutes provide a moral right of integrity against altering a work or making a work available that is offensive to the author or violates the work’s reputation or individuality; the Swedish provision is also subject to a more general requirement that the work “not be altered to a greater extent than the use requires”.^84^ The Dutch statute provides both a moral right of integrity against “distortion, mutilation or other impairment of the work that could be prejudicial to the reputation or name of the maker” and a right against all alterations, unless opposition to the alteration “would be unreasonable”.^85^ Quotation under German law is subject to a prohibition of alteration.^86^ The absence of an “extent” threshold or a prohibition limited to alterations impairing the work or harming the author means that, in this respect, the German quotation permits a narrower range of uses although the prohibition of alteration should not be applied in such a very strict manner as for example in academic research.^87^ However, the overall wiggle room for those who quote remains unclear. The German quotation exception also expects a deliberate, purposeful dialogue between quoted and quoting work, a dialogue which is enabled in part by unaltered quotes. The appearance of the quoted material must be recognisable to listeners as a quotation (which tends to limit the quotation exception to the reuse of well-known musical material, especially with a lay-listener standard as is common in Germany). This prohibition materially narrows the usefulness of the German quotation concept, as alteration is common in many traditions, genres and practices of reuse, both in music and other arts.^88^

#### German Restrictions Regarding Music

Finally, we note that the German quotation concept is subject to an important restriction regarding music. As with the German free use, melodies are also excluded from the German quotation exception, taking away an appropriate and straightforward scenario for lawful musical quotation.^89^ However, this provision would be repealed by the proposed German reforms implementing the DSM Directive.

### Gaps Between National Free Use and Quotation Provisions

While we have set out commonalities between national free use provisions, a comparison between national provisions for free use and quotation provisions reveals several significant differences in what they permit. In short, national quotation provisions provide less permission for unlicensed yet lawful reuse of protected material than free use exceptions, though the reasons for the gap vary between jurisdictions.

#### Purpose and Extent

Perhaps the most extensive gap between national free use and quotation provisions, at least where reuse in the arts is concerned, relates to the purpose and extent of quotation. While the Dutch quotation provision explicitly lists acceptable purposes for lawful quotation, the German, Norwegian and Swedish quotation provisions also implicitly require a purpose (to which the extent of allowable quotation is tied). The fact that these quotation provisions require a purpose – which could not be quotation itself, for such an interpretation would render the word “purpose” meaningless – creates a hurdle not present in the free use provisions. Whereas a person relying on free use can do so without any clear, intended or discernible purpose, a person hoping to rely on these quotation provisions would need a purpose and, where German, Norwegian or Swedish law applies, quote only as much as the purpose justifies.

#### Acceptable Alterations

A further gap is the concept of good practice (or social custom), which applies to quotation but not free use. Uses that are not in accordance with good practice may be free use, but not quotation. At least under Dutch, Swedish and Norwegian law, this requires an appropriation artist or other creator hoping to rely on a quotation provision to consider how to stay within the bounds of the relevant practice (or custom).

Furthermore, the overall intent of free use provisions is to allow transformative reuse (to allow fading/sufficient distancing) while transformativeness is not the main characteristic of a quotation. To the contrary, quotations are interested in the presence of the quoted material for a specific purpose. Fading would be counterproductive here. Quotation draws its power from using something significantly unaltered. In this regard, free use and quotation are opposing aesthetic and legal concepts.

#### Requirement of Independent Work

Where a user does not create an independent work, quotation might generally be preferred over free use, which is available only to creators of independent works. Quotation provisions in Norway, Sweden and the Netherlands do not require creation of an independent work, though this is not the case in Germany. However, in the context of reuse in the arts, where reuse typically yields an independent work, it is unlikely that the requirement of an independent work would be an impediment to free use.

Due to such differences, we consider that foregoing free use provisions would be a dramatic and far-reaching change of system. Especially where national quotation provisions impose limits on permissible purposes, the extent of quotation and the range of acceptable alterations, there are significant hurdles for potential users of copyright and downstream creators. As we will explain, the gap between national free use and quotation widen further when EU law requirements are imposed on national quotation provisions.

## Gaps Between EU Law and National Requirements on Quotation

This section aims to describe the InfoSoc quotation requirements in the reading of the ECJ and draw out where and how national quotation provisions meet with or deviate from those EU requirements. As in the previous section, we focus on comparisons with four free use countries (Germany, the Netherlands, Norway and Sweden). We consider both where national provisions are potentially consistent with EU law and where EU law imposes requirements additional to national provisions.

### Where National Provisions Are Potentially Consistent with EU Law

Before considering gaps, we set out several areas where national provisions are potentially consistent with EU law. While the statutory wording may vary between national and EU provisions, provisions may remain consistent where textual variations have less material impact.

#### Extends to Use of Works and Other Subject Matter

The InfoSoc Directive allows member states to extend a quotation exception or limitation to the use of not only works but also performances, phonograms, films and broadcasts, as described in Arts. 2 and 3. As discussed in the previous section, national quotation provisions permit quotation of a range of copyright and related rights subject matter, though the exact range varies. The Swedish statute extends the quotation provision for works to also carve out of related rights in Chapter 5. Likewise, the Norwegian statute extends the quotation provision for works to also carve out of related rights in Chapter 2. Regarding most related rights, the German Act includes in the respective provisions an explicit extension of the quotation exception to the respective related right. The Dutch quotation provision covers a range of materials; although the statute mentions only “the copyright in a literary, scientific or artistic work”, the official preparatory document accompanying the amendment explains that the provision can permit “sound quotes” (*klank-citaat*), amongst quotes of other materials.^90^

#### Attribution Unless Impossible

The InfoSoc Directive quotation provision requires attribution “unless this turns out to be impossible”.^91^ None of the national provisions discussed in the previous section replicates the InfoSoc language exactly, but it should not be assumed that they conflict with InfoSoc in the absence of authorities to the contrary. Germany most closely mirrors InfoSoc, by making attribution the default requirement unless the quoting person does not know the source. The Norwegian and Dutch provisions are less close, only requiring attribution where practically or reasonably possible; likewise, the Swedish provision only requires attribution in the manner required by good practice.

#### In Accordance with Fair Practice

The InfoSoc Directive quotation provision permits only uses that are “in accordance with fair practice”.^92^ As discussed in the previous section, the Norwegian, Swedish and Dutch provisions materially implement this by permitting only quotation in accordance with good practice or acceptable social custom. The German statute has no such statutory requirement, though the case law clearly reflects this requirement, too, related to conflicting fundamental rights and the need to balance them.

### Where EU Law Imposes Requirements Additional to National Provisions

However, there are a range of gaps between national quotation provisions and requirements in EU law, as well as areas with small, potentially immaterial gaps. While some of the gaps exist in statute alone, how courts interpret EU and national quotation provisions also has a material influence. Additional gaps occur that further minimise the potential of quotation provisions to work as an effective surrogate for free use provisions that allows the member states to maintain a similar space for unlicensed yet lawful reuse in the arts as before.

#### Purposes Narrowed to “Such as Criticism or Review” and Dialogue Requirement

An obvious gap between EU and national provisions is the InfoSoc limit on purposes for quotation. The InfoSoc Directive limits quotation exceptions to those which are for “purposes such as criticism or review”.^93^ As discussed in the previous section, three of the countries under investigation here with free use and quotation provisions (Germany, Norway and Sweden) have no such requirement.^94^ The Dutch provision restricts quotations to “an announcement, review, polemic or scientific treatise or a piece with a comparable purpose”,^95^ and a Dutch court has considered one type of quotation for a purpose outside of “such as criticism or review”. The Court of Appeals of Arnhem considered search engine results (including text property descriptions, addresses, prices and photos of the properties) from online real estate databases to be such quotations.^96^ However, despite the absence of “purposes such as criticism or review” in these cases, it remains unclear exactly how much the national provisions are in conflict with InfoSoc. National court interpretation of national quotation provisions may affect their scope, and there is no authority establishing how far from “criticism or review” is permitted by the InfoSoc language “such as”.^97^

“Purposes such as criticism or review” may be well suited to quotation in academic research or journalism but are obviously too narrow for quotation in the arts, especially when considered in tandem with the ECJ’s dialogue requirement in *Pelham*. This requirement limits lawful quotation in the arts solely to those with the intention of “entering into dialogue”, which includes those with “the purposes of illustrating an assertion, of defending an opinion or of allowing an intellectual comparison between that work and the assertions of that user”.^98^ This dialogue requirement was previously rejected, for example, by the German Constitutional Court in *Germania 3*, which recognised the lawfulness of quotation in the arts for the purpose of deepening its own aesthetic message and where the quoted material becomes a mere design tool (“*Gestaltungsmittel*”) for the creation of the new work that an artist reuses solely for its aesthetic qualities.^99^ The *Pelham* decision, particularly the dialogue requirement, prevents such a use from being lawful under EU law.^100^

Further complications arise from the fact that the requirements for purpose and dialogue are mixed up to some degree. The ECJ opined in *Pelham* that the user relying on a quotation must have the intention of “entering into dialogue”.^101^ This is, in a sense, also part of the purpose of a quotation.^102^ The ECJ added in *Pelham* that “there can be no such dialogue where it is not possible to identify the work concerned by the quotation at issue”.^103^ This, in effect, creates a requirement that the quoted work is identifiable in quoting a work. It is unclear how this concept of “not possible to identify the work” will apply.^104^ This seems to present a challenge for short, obscured or otherwise modified quotations, which would be difficult to identify. For example, it may not be possible to identify the sampled work when the sample is merely of a single drum kick or a split second of an instrumental track. However, it may be possible to identify the quoted work where there is attribution, which may be the majority of cases. The InfoSoc Directive quotation provision requires attribution unless impossible. In cases where there is attribution, it seems that the ECJ’s requirement to identify the work will also be met, notwithstanding that attribution is *inter alia* a statutory condition on lawful quotation deriving from InfoSoc and identification is an ECJ-made condition on lawful quotation.

It is also worthwhile to comment on the nature of the dialogue requirement. It appears that the dialogue requirement elaborates only on “quotation” and not “purposes such as criticism or review”. That said, certain musical quotations may satisfy both “entering into dialogue” and “purposes such as criticism or review”, especially if the quotation use or the work of quotation deals with events happening now or events of current interest. Songification of news, which turns news videos into music videos by vocoding speech into melodies and adding backing instrumentals, provides several examples. Pogo’s *Trumpular*, which combined quoted utterances of the then presidential candidate Donald Trump with musical quotes from *Upular* (itself a mashup of audiovisuals from the Disney film “Up”), is another. By focusing on the “intention of entering into dialogue”, the ECJ establishes a subjective standard. The intent, not the outcome, would be the focus. It is not clear how the user’s intention can be established, but the test does not seem to set an objective standard, which would require the user to actually enter into dialogue with the used material.

In sum, the ECJ appears to add to national quotation requirements for the states we investigate here which are not limited to the specific purposes and are not clearly subject to a dialogue requirement.

#### The Extent Required by the Specific Purpose

Beyond the purpose itself, the InfoSoc Directive also limits quotation “to the extent required by the specific purpose”.^105^ All four national statutes we consider have a provision which complies with this InfoSoc requirement. However, in *Spiegel Online*, the ECJ invoked the Berne three-step test to set another limit on the extent of quotation which does not exist in EU jurisdictions with free use and quotation.^106^ It cited Art. 5(5) of the InfoSoc Directive to rule that

the use of the quoted work must be secondary in relation to the assertions of that user, since the quotation of a protected work … cannot be so extensive as to conflict with a normal exploitation of the work or another subject matter or prejudices unreasonably the legitimate interests of the rightholder.^107^

The *Spiegel Online* approach is more permissive towards a quotation that yields a work that the rightholder of the quoted work would not typically create, but less permissive to standalone quotations. An example of standalone quotations is the burgeoning practice of creating sample libraries from copyrighted recordings and musical works, which may now fail to qualify for an exception and require more licensing.

Proving that a use is secondary to the user’s assertions is a challenging test for musical quotation and musical appropriation, especially where there are no lyrics in the musical work or phonogram. What kind of melody, rhythm or harmony asserts something different from the quoted material? What combination of timbral, performed or studio-engineered aspects help a phonogram assert something different? Assertion might be particularly difficult to establish with respect to sound recordings. Whereas musical works benefit from notation, sound recordings lack a standard language for describing extent. Waveform notation and digital audio workstation timelines are hardly a basis for establishing that a use is secondary to the user’s assertions.

Still, some musical quotations may fare better at satisfying these tests. The Berne Convention’s optional quotation provision lists two such types: quotations from “newspaper articles and periodicals in the form of press summaries” and “illustration for teaching”.^108^ For example, songification of news, where spoken news dialogue is converted into melody and with accompanying instrumentation, may quote from news to form a particular type of press summary. Likewise, educational online videos which quote phonograms to depict a particular genre or era of music may quote music to illustrate for teaching. But overall, these are rather specific acts of musical reuse. For most cases, this line of argument of the ECJ provides additional challenges and limitations.

#### Quoted Subject Matter Lawfully Made Available to the Public

The InfoSoc Directive limits quotation to use of works “lawfully made available to the public”.^109^ As the previous section explained, the Dutch provision includes this limitation but the German, Norwegian and Swedish provisions require only that that the quoted work is published, regardless of the lawfulness of publication.^110^ This is a material gap, especially in the context of online content where the origins of a copy are often difficult to ascertain, even outside of cases of orphan works.

## Possible Arguments for Bypassing ECJ Interpretation of Exceptions and Limitations

In the previous sections, we have established the diagnosis of considerable gaps between national free use and national quotation provisions, as well as further gaps between national quotation provisions and EU quotation requirements. If national copyright is reformed, as we expect, to reduce permission for unlicensed yet lawful reuse mainly to these quotation purposes and requirements, this would indeed represent a significant loss for unlicensed yet lawful reuse in the arts and those engaged in cultural borrowing.

Those who share our view (see Sect. 5) that this result is undesirable and inappropriate may challenge it from different directions in the hope of avoiding it. As mentioned in the introduction, there are several arguments in the discourse for bypassing and averting these consequences of *Pelham* (and the other recent ECJ judgments). In this section, we explain why we do not believe, given the present position of the ECJ, the four main arguments succeed in addressing the problems resulting from the gaps we identify between free use and quotation provisions. As engaging as these four arguments are and as welcome as we find their intended outcomes, we see them as being, at best, long shots in light of *Pelham*, *Spiegel Online*, and *Funke Medien*. The ECJ’s choices to bind together three cases from the area of reuse (albeit with only *Pelham* from the field of arts) and to sit as a Grand Chamber give the positions set forth in these cases stability, coherence, and longevity. In our view, these choices inhibit an easy or likely overturning of these positions.

### Harmonisation in Relation to EU Rights, Limitations and Exceptions, and Room for National Discretion

The first argument questions whether the ECJ’s position can be interpreted in such a way that national free use provisions could nonetheless move forward by way of national discretion regarding the requirements of the InfoSoc Directive. The position prior to *Pelham* was that free use was in fact a specific, privileged form of adaptation that was otherwise subject to the licensing obligation. Free use understood this way did not conflict with the InfoSoc reproduction right, noting the InfoSoc Directive contains no adaptation right. Yet, *Pelham* clearly involves a case of adaptation and the ECJ puts effort into defining under which circumstances (specifically, unrecognisability) such an adaptation does not infringe the reproduction right. The pre-*Pelham* position becomes unsustainable in the light of *Pelham* – besides scenarios when adaptation does not also involve at least some reproduction – for several reasons.^111^ Firstly, reproduction rights are fully harmonised.^112^ Secondly, reuse as we are dealing with here – reuse of protected parts (sufficiently original)^113^ of a third-party work – involves reproduction in a legal sense in every case.^114^ And thirdly, *Pelham* is not to be understood to be restricted to cases of Art. 2(c) InfoSoc only, but to encompass all reproduction rights, as the German Federal Court of Justice recognised both before and in its direct follow-up decision to *Pelham*.^115^ So, this position of perpetuating the respective national free use provisions by bypassing the quotation provisions and the challenges of the gaps between both can only proceed if there is wiggle room for national discretion despite *Pelham*.

Harmonisation in EU copyright law places different constraints on national discretion regarding national copyright law. EU (and EEA) states have little discretion as regards InfoSoc rights for reproduction, communication (and making available to the public) and distribution, as the ECJ has confirmed in *Pelham*, *Funke Medien* and a number of earlier decisions.^116^ However, the situation is indeed more complex regarding harmonisation of EU limitations and exceptions. Here might be a starting point for this position, arguing for continuation of free use provisions.

On the one hand, in *Pelham*, the ECJ opines “a Member State cannot, in its national law, lay down an exception or limitation other than those provided for in Article 5 of Directive 2001/29 to the phonogram producer’s rights provided for in Article 2(c) of that directive”.^117^ This confirms that member states have no discretion to provide free use as a limitation or exception to InfoSoc rights to the extent they exceed Art. 5 exceptions or limitations. In addition, in *Spiegel Online*, the ECJ opined that “Member States may provide, in their law, for an exception or limitation referred to in Article 5(2) and (3) of Directive 2001/29 only if they comply with all the conditions laid down in that provision”.^118^ This constrains member states from providing national exceptions that do not comply with all of the InfoSoc (and any other EU law) conditions for Art. 5 exceptions and limitations. As we have laid out earlier in this article, many national exceptions deviate from such InfoSoc conditions. If this constraint is applied strictly, member states would not have discretion to deviate in this manner.

On the other hand, in *Funke Medien*, the ECJ clarifies that provisions of an EU directive “‘allow [some] discretion in terms of implementation in national law’, provided that that condition is understood as referring to the degree of the harmonisation effected in those provisions, since such an application is conceivable only in so far as those provisions do not effect [sic] full harmonisation”.^119^

From such precedents, some legal scholars have noted that there might exist considerable wiggle room for discretion for member states.^120^ If so, that might be an option to retain the free use provisions. In this sense, we align with Geiger and Izyumenko who argue that the quotation exception holds “potential for a flexible interpretation”, even if it falls short of what a free use limitation provides.^121^ In the previous section, we highlighted some instances where national provisions are potentially consistent with InfoSoc requirements, even though they might deviate from the exact language. For example, a national attribution requirement qualified by “where practically possible” might be sufficiently close in practice to the InfoSoc requirement of “unless impossible”. Likewise, a national requirement that quotation is in accordance with “social custom” that is “reasonably acceptable” might be consistent with the InfoSoc requirement of “fair practice”. Member states may also retain some modest discretion regarding the permissible purposes for quotation. Given that InfoSoc specifies “purposes such as criticism or review”, it is clear that not requiring a purpose at all – which is the approach of the German, Swedish and Norwegian statutes – exceeds the discretion member states have under EU law. However, it is possible that member states have discretion to permit a number of purposes, as long as they relate to criticism or review. This remains, fundamentally, untested ground. If “such as” only permits purposes extremely close to “criticism” and “review”, this permits minimal reuse in the arts, given that review is less relevant to reuse and that reuse for the purpose of criticism is already (at least partly) covered by parody and caricature under InfoSoc Art. 5(3)(k). The requirement to address the source could also be relegated to accompanying material and still be addressed sufficiently, as is necessary in non-textual arts.^122^

However, as the *Spiegel Online* and *Funke Medien* decisions explore in detail, in the ECJ’s mind the member states’ discretion is severely circumscribed in several regards. Member states are not free to determine the parameters governing the exceptions and limitations, and must follow their respective objective without going beyond what is necessary to achieve it. Moreover, member states must not endanger a high level of protection and a proper functioning of the single market.^123^ As is also highlighted by the ECJ in *Pelham*,^124^ taking into account that free use provisions are uncommon in the EU, both objections provide significant obstacles to free use provisions, which have (or could) overcome other ECJ requirements (such as the Berne three-step test in the German Constitutional Court’s *Pelham* judgment).^125^

In the end, as Jütte rightfully notes, this discretion provides little flexibility for member states, arguing that limitations and exceptions are “subject to a strict interpretation that limits their flexible application”.^126^ Engman, similarly to Jütte, explains that through its *Pelham*, *Spiegel Online* and *Funke Medien* decisions, the ECJ “makes it doubtful whether there is de facto any national discretion in regard of exceptions and limitations”.^127^ Insofar, we follow Rosati’s position:

First, the court confirmed that the EU copyright framework does not leave Member States the freedom that, clearly, they thought they had. Case law issued over the past few years has been consistent in this sense, and the holding in *Pelham* is not different. The EU copyright framework is an (in)flexible, rather than a flexible, one.^128^

### Consideration of Fundamental and Human Rights

A second argument is that fundamental and human rights can assist in narrowing the gap between national free use and national quotation provisions. From this point of view, and in the context of unlicensed yet lawful reuse of protected musical works, performances, and media, restrictions to the rights protected in Arts. 2 to 4 InfoSoc Directive are limitations to the right of property (Art. 17 CFR) in the service of the freedom of expression (Art. 11 CFR) and the freedom of the arts (Art. 13 CFR), all fundamental rights.^129^ There are several prongs to this argument.

Firstly, the recent ECJ judgments indeed recognise several links between EU fundamental rights and copyright and related rights. Through this, they seem to open the door to a narrowing of the rights granted and protected in Arts. 2 to 4 InfoSoc beyond the question of exceptions and limitations based on fundamental rights.^130^ The ECJ recognises, for example, cases where sound sampling is not a reproduction in legal terms under Art. 2(c) of InfoSoc insofar as a user is “exercising the freedom of the arts”, which it links to freedom of expression.^131^ However, this turns out to be very limited wiggle room that is reduced to cases of unrecognisabilty. Beyond this, the ECJ’s recent judgments do not justify expecting a further narrowing of rights through fundamental rights. Quite the contrary, the ECJ *Pelham* judgment rejects such an approach of both the German Federal Court of Justice and the German Constitutional Court to apply the German free use provision to all reproduction rights including phonogram rights on the basis of fundamental rights.

Secondly, the recent ECJ judgments seem to open the door to a broadening of InfoSoc’s Art. 5 exceptions and limitations, too,^132^ based on EU or even national fundamental rights.^133^ In *Pelham*, the ECJ privileges quotations which invoke the freedom of expression and to a less-pronounced extent, freedom of the arts.^134^ At the same time, however, the ECJ explicitly rejects national discretion to retain the much broader free use provisions outside of the InfoSoc framework.^135^ The ECJ did not reject the possibility that the EU legislature might amend the InfoSoc exceptions and limitations with such free use provisions, as some commentators hoped for. However, the ECJ has made no such amendment. Furthermore, the interpretation of national fundamental rights also cannot be in conflict with the ECJ’s interpretation of EU fundamental rights.^136^ So, practically, there is no way to bypass the ECJ’s position which is much more constraining regarding the possibility of broadening the wiggle room of exceptions and limitations in favour of reuse in the arts as, for example, the German Federal Court of Justice and the German Constitutional Court accepted earlier in *Pelham*. Thus, as was the case with the narrowing of the scope of InfoSoc’s rights, the actual leeway for broadening accepted by the ECJ is very limited. In this regard, we follow the position of van Deursen and Snijder:

[Advocate General Szpunar’s] opinion thus points towards permitting an external role for fundamental rights in particular cases... However, as evidenced by its judgments, the CJEU does not want to give into this theoretical possibility and does not hint towards fundamental rights playing any role that exceeds their use as yardsticks for the interpretation of the provisions of Art. 5.^137^

In Sect. 4.4, we explain other reasons against broadening InfoSoc’s quotation provision.

Thirdly, one can argue that fundamental rights function as an external restriction to copyright and can thus change the outcome of the balancing between the respective fundamental rights involved.^138^ It is true that the scope of EU fundamental rights must be interpreted in accordance with each other,^139^ and a “fair balance” must be achieved.^140^ In sum, the interpretation of the EU directives (like the InfoSoc Directive and DSM Directive) must be compatible with this scope.^141^ However, the InfoSoc legal regime, including InfoSoc exceptions and limitations, is itself already the result of legislative balancing between the fundamental rights.^142^ EU fundamental rights are not an additional, external test to be applied to the InfoSoc Directive.^143^ In the context of reuse in the arts in *Pelham*, the ECJ’s balancing of fundamental rights has occurred within the InfoSoc legal regime, in the interpretation of InfoSoc’s rights as well as exceptions and limitations.^144^ In doing so, the ECJ engaged here in the kind of explicit balancing act it refrained from in *Deckmyn*, regarding parody.^145^

Overall, it seems unlikely in the light of the current ECJ position to expect that fundamental and human rights can assist in narrowing the diagnosed gaps between national free use and national quotation provisions.

### National Statutory Reform Implementing the DSM Directive as a Basis for an Alternative Strategy to Perpetuate Free Use Provisions

While under the InfoSoc Directive, national states held discretion to introduce optional quotation provisions, this discretion gives way to mandatory quotation provisions for online content-sharing uses under the DSM Directive. At the time of writing, the EU member states have less than one year to implement the DSM Directive provisions, a current process that is no less contested and controversial than the legislative process for the DSM Directive.^146^ As a result, in parallel with the ECJ’s interpretation of the InfoSoc Directive, the DSM Directive will place further pressure on member states to align their laws, by making quotation, criticism and review (as well as parody, caricature and pastiche) exceptions or limitations mandatory on EU (and likely EEA) member states in regard to online content-sharing services (such as YouTube, SoundCloud and Spotify).^147^ This will require member states to address gaps between national quotation provisions and EU law provisions. Where a national provision conflicts with the InfoSoc Directive, national statutory reform may be the best course of action to ensure national laws reflect EU requirements, for example to limit purposes beyond “purposes such as criticism and review” (where this is not already the case). Without statutory reform, a careful, black-letter reading of the national statute may mislead practitioners and copyright stakeholders. Where it is less clear about national conflict with the InfoSoc Directive, it may be prudent to defer statutory reform. This would allow time for judicial interpretation of existing statutes, in light of the ECJ interpretation of the InfoSoc Directive, and preserve an option for future statutory reform. This may be the case, for example, in the countries where the attribution requirement for a quotation provision is “where practically possible” rather than “unless impossible”.

However, this will not only require member states to address gaps between national quotation provisions and EU law provisions, but also offer room for alternative strategies to attempt to salvage some former free uses, especially through the pastiche exception which is still untested before the ECJ. The German government acts as a frontrunner in this regard. We note the German government has already proposed statutory reforms to address not just the DSM Directive but explicitly also the aforementioned recent ECJ interpretation of EU copyright and related rights. In our view, the German proposal severely tests the limits of national discretion. The proposal tries to reintroduce some of the substance of the former free use provision through two other provisions: limiting the adaptation right (proposed Sec. 23 of German Copyright Act) to uses without sufficient distance (“*kein hinreichender Abstand*”, following the intention of question 3 of the German court in *Pelham*) and introducing a broad pastiche exemption (proposed Sec. 51a) to include amongst other things: “Remix, Meme, GIF, Mashup, Fan Art, Fan Fiction, Cover und Sampling”.^148^

Arguably, amending an adaptation right does not conflict with the InfoSoc reproduction right, noting the InfoSoc Directive contains no adaptation right, yet it is difficult to envision an adaptation that does not also involve some reproduction which would lead back into the ECJ’s framework on how to handle rights as well as exceptions and limitations in the InfoSoc Directive.^149^ In *Pelham*, the ECJ only excludes cases where the reuse is unrecognisable from infringing Art. 2(c) InfoSoc Directive. Yet, these are conflicting concepts, as sufficient distance is not unrecognisability. To the contrary, “sufficient distance” allowed the original still to shine through in the “free adaptation”.^150^

We also note a pastiche exception is specifically permitted by the InfoSoc Directive and mandated on member states by the DSM Directive. However, its scope remains untested in courts, even as it gains momentum in the scholarly literature to be interpreted in broad terms.^151^ However, we do not expect the German government’s proposed interpretation of pastiche, so broad that it approaches free use, would survive the first contact with the ECJ.^152^ Amongst other things, it runs against the legal and aesthetic history of the term, the handling of the pastiche exception in other member states, and the EU’s deliberate decision not to introduce a free use exception through the DSM Directive but rather the pastiche exception instead. It also goes against the close relationship implied between pastiche, caricature and parody, all found in Art. 5(3)(k) InfoSoc Directive, suggesting a similar, defined purpose and scope. Furthermore, the result lacks – in stark contrast to the former free use provision (sufficient distance)^153^ – necessary and sufficient conditions to determine what kind of pastiche reuse in the arts will need a licence and what goes free.^154^ In the absence of such conditions, a pastiche exception may not fulfil the “certain special cases” prong of the three-step test requirement of InfoSoc and the ECJ. Furthermore, we see pastiche as being separate from quotation and not, as Aplin and Bently have argued recently, a mere sub-group of quotation.^155^ In our understanding, pastiche only applies in cases of imitation of the specific stylistic features of a genre (which does not engage InfoSoc rights) and especially the personal style of an author or a work (which can infringe – and has in past decisions – reproductions rights as well as moral rights).^156^ Although some pastiche involves direct reuse of protected expression, direct reuse is not a necessary and sufficient condition of pastiche. By contrast, pastiche must involve imitation. Due to this narrower understanding of pastiche, we expect that national pastiche exceptions in member states will not compensate for the loss of free use provisions for unlicensed yet lawful reuse in the arts.^157^ Although pastiche has been an optional exception since the InfoSoc Directive in 2001, it has been conspicuously absent from the legal scholarship on the *Pelham* litigation since the first court decision in 2004. As such, it is surprising that the German government’s proposed copyright reform now deems “sampling” to be an example of pastiche.^158^

### Expansive Broadening of InfoSoc’s Quotation Provision

At the outset, we stated our view that the quotation provisions provide the best chance for unlicensed reuse in the arts to be determined legal. However, in Sects. 2 and 3, we outlined the severe EU and national limits on the scope of the quotation provisions that prevent them from fully replacing the free use provisions in the four countries analysed here with regard to reuse in the arts. Naturally, broadening the quotation provisions to push past and around these limits would help to bridge the gaps between the free use provisions and the quotation provisions. Here, Jütte and Maier (based on fundamental rights, as we discussed in Sect. 4.2) as well as Aplin and Bently (based on a close reading of the Art. 10(1) Berne Convention and its history) lead the charge in exploring a much broader understanding of quotation free from most EU and national limits, which would leave no significant gaps between free use and quotation provisions. This is an alternative attempt to reach a result similar to a broad pastiche exception (as we discussed in Sect. 4.3). Aplin and Bently list features of quotation that, in their view, enable a scope far beyond what EU law permits:

If it was necessary to identify any necessary features that render a specific act one of “quotation”, it would be that (i) the quotation involves the reuse of expressive material (ii) for its expressive qualities, where (iii) that material is recognisable, or could be recognised, as material authored by another and (iv) is used or intended to be used in an act of expression, or at least in representation, for its expressive qualities.^159^

As Bently and Aplin argue, the Berne quotation provision is mandatory and “should not be subjected to additional conditions beyond those recognised in Art. 10(1). To do so is to breach the obligation”.^160^ Generally, Berne provides a minimum level of protection, meaning that signatory states comply with Berne if they provide the full suite of rights but choose not to introduce optional Berne exceptions.^161^ However, as Bently and Aplin argue, the Art. 10(1) quotation right is mandatory, starting with “[i]t shall be permissible to make quotations”, contrasting with “it shall be a matter for legislation in the countries” in Arts. 9 and 10^bis^, which makes clear that countries hold the choice of introducing such limitations or exceptions.^162^

There are legal arguments against Aplin and Bently’s broad concept of quotation, at least in the context of EU law and reuse in the arts. Foremost, the ECJ’s *Pelham* decision, including its interpretation of InfoSoc requirements of quotation, binds EU states to a much more limited scope, at least for the time being. Even Aplin and Bently concede how difficult it would be to enforce their interpretation of Berne, which has not been implemented for more than half a century.^163^ In an EU law context for reuse in the arts after the recent ECJ decisions, we simply cannot ignore the ECJ requirement for a stricter understanding of quotation as an autonomous concept of EU law. It is true that the interpretation of the InfoSoc quotation provision needs to be interpreted in a way “that enables its effectiveness and safeguards its purpose”.^164^ However, there is some room left for effective, if targeted, application of the quotation provision for cases of reuse in the arts even if significant requirements are attached to it as the German Constitutional Court decided in *Germania 3*.^165^ It is just that this room is smaller compared to free use provisions. That does not mean it is ineffective (as *Germania 3* showed) but different in comparison. This is true even if one follows our view that under EU law the dialogue requirement has to be added to the requirements set in *Germania 3*. However, many typical acts of reuse in the arts have no problem with this requirement, compared to other requirements (e.g. strict limits to extent by purpose, prohibition of alteration, or acknowledgment of source).

Beyond this, we see several other arguments against such a broad reading.^166^ The first has to do with the conception of quotation itself, as Parkin notes.^167^ If quotation provisions were more or less the same as free use provisions, why would countries include both in their statutes?^168^ As we showed in Sect. 2, the concepts of quotation and free use differ in each of the four jurisdictions we considered and cannot replace one another. That quotation, despite existing before InfoSoc, is not highlighted as a possible exception in the legal scholarship on the *Pelham* litigation also favours a narrow, not broad reading.^169^ This is especially striking because the sampling in *Pelham* is a rare, pure quotation in music: free of other layers of music, only marginally transformed and therefore clearly recognisable at the beginning of the defendant’s track.

Secondly, this broad interpretation of quotation fails to provide the necessary and sufficient conditions to distinguish whether reuses in the arts are lawful quotation. Getting rid of requirements such as strict limits to extent by purpose, prohibition of alteration and acknowledgment of source means getting rid of the most characteristic qualities of quotations compared to other forms of adaptation.^170^ For example, the suggestion is correct that the requirement to address the source has to be handled differently in cases like reuse in music where devices like quotation marks are not suitable. Regarding non-textual arts, the InfoSoc quotation exception has to be interpreted extensively to make sense.^171^ However, there are many ways in music to indicate that it is third-party material one is using.^172^ Without such requirements, quotation becomes a mere synonym for borrowing, which it is not. Such a broad reading is not useful for the acceptance of copyright law in the arts, and stands in stark contrast to the InfoSoc and ECJ obligation that exceptions and limitations are limited to certain special cases, as is the extent of third-party material used in each act of quotation to the specific artistic purpose.^173^

Thirdly, such a broad understanding does not seem to have been intended in the Berne Convention. There were alternative legal concepts available prior to the 1971 Paris revision of the Berne Convention that were clearly broader than quotation but were not taken up; for example, the current German free use provision had been in force since 1966 and its predecessor dates back to 1902. Quotation as a term would be a rather odd choice to achieve a free use provision as the four nations analysed here show. Fourthly, if quotation has such a broad meaning, separate exceptions for parodies, caricatures, and pastiches would be redundant.

Due to these reasons, we follow Rosati’s position:

The EU copyright framework is an (in)flexible, rather than a flexible, one. Secondly, the court had the chance to elaborate a bit further on the concept of quotation (it first tackled it in *Painer*, C-145/10, EU:C:2013:138) and clarified that, indeed, a quotation cannot be for any reason but rather needs to satisfy a number of conditions for the exception to apply.^174^

## Conclusion

In retrospect, the timing of the ECJ decision proved to be unfortunate, coming just months too late to influence the DSM Directive’s legislative process. In our view, the DSM Directive offers to nations with a free use provision a striking lack of provisions which either accommodate contemporary reuse or pre-emptively compensate for the ECJ’s narrowing of exceptions to those specified in the InfoSoc Directive.^175^ In the field of reuse, the DSM Directive mandates only two InfoSoc Directive exceptions – one for caricature, parody and pastiche, and another for quotations “for purposes such as criticism or review” – for certain online-platform uses, though countries are free to extend this to all uses, as the current German legislative proposal does.^176^ No new exceptions and limitations were introduced. This was a surprising missed chance to update EU laws for the digital age of retromania, remix culture, user-generated content, and social media, given that the InfoSoc Directive limitations and exceptions are the result of a legislative process in the 1990s.^177^ And it is a missed chance for which artists and the accompanying cultural industries are now paying very dearly in the light of *Pelham* which followed soon after.

But, for the time being, we see quotation provisions as the main mechanism for salvaging at least some flexibility lost with free use provisions. Following the recent ECJ copyright decisions, the unlicensed yet lawful reuse in the arts becomes more reliant on quotation provisions, in spite of their many requirements. This is especially true online where social media and online communities have created a participatory environment with creative reuse at its centre, but copyright licensing does not enable lawful reuse in many cases.^178^ To us, it seems realistic to expect that member states and additional participants in the single market must rely on quotation provisions for now when it comes to unlicensed yet lawful reuse in the arts for all cases that do not belong to the relatively and aesthetically narrow concepts of parody, caricature, or pastiche. This poses a huge challenge for the many forms of reuse in the arts that are not mocking or humorous and therefore would not qualify as parody under *Deckmyn*.^179^ For forms as varied as posts, retweets, anime music videos, memes, GIFs, remixes, and mashups, quotation provisions will become an essential ingredient for professional and user-generated content alike. However, if lawful quotation is limited, for example, to uses entering into dialogue and those where the quoted work remains identifiable, and content identification fails to recognise even this narrowed range of quotations, it is certain that reuse will come into greater conflict with EU and EEA national laws – including the fundamental freedoms of expression and of the arts. This is a challenge especially for contemporary music where deliberate appropriation is widespread for diverse important aesthetical, cultural, and social reasons. The new, post-*Pelham* copyright regime does not suit this musical present. Reusers in the arts, other than financially potent superstar artists and corporations that can afford a licence, will have to adjust their artistic practices if parody, caricature, and pastiche, unmodified minor quotations and modifications to the point of unrecognisability will be all that is lawful. Such a new status quo would constitute a huge loss of cultural production, aesthetical diversity and social participation to creators in the countries which will now lose their free use provisions.

By setting out the gaps between quotation provisions in the face of the present national and EU requirements and former free use provisions, we generate additional arguments in the legal discourse for introducing free use (or similar provisions) into the catalogue of EU exceptions and limitations. In our view, this is needed now, especially because an expansive reading of quotation (or alternative concepts like pastiche) is not possible in our understanding within the InfoSoc Directive’s legal framework and is at least not likely to take place given the present position of the ECJ. To suggest such a legal reform is, of course, not new.^180^ However, addressing it has become even more urgent after *Pelham*, at least for the discussed countries with broad free use provisions.

Furthermore, highlighting the gaps and the insufficiency of the already given alternative EU limitations and exceptions is important to help artists as well as the accompanying cultural industries to avoid the erroneous assumption that other pre-existing exceptions and limitations will do the job of free use, and avoid risky and arguably fruitless appeals to the ECJ for a broad interpretation of these limitations and exceptions. The recent German experience with *Pelham* which forced domestic copyright reform regarding the requirements for unlicensed yet lawful reuse should be a warning. The matters addressed by this reform could – and should – have been clarified before the first German federal *Pelham* decision in 2008. The ECJ’s judgment more than a decade later in 2019 brought a considerable and likely (if not foreseeable) legal change regarding the lawfulness of not just future reuse but also a past generation of artistic reuse stretching back to 22 December 2002.^181^ Such legal uncertainty undermines many reuse practices that are essential to music-making in general.

Salvaging unlicensed yet lawful reuse is a significant challenge that would ultimately need to be addressed by legislators. This is especially true from the perspective of countries which operated with broad free use provisions. In the absence of EU legislative reform, we expect continuing disputes over the breadth of the EU quotation and pastiche concepts, as well as the ability to retain national free use provisions in the hope of enabling unlicensed yet lawful artistic reuse. As we have explained in this paper, however, we believe such disputes have at best a slim chance of succeeding under the ECJ’s now forcefully confirmed narrow reading of InfoSoc’s rights as well as exceptions and limitations. In the light of *Pelham*, *Funke Medien*, and *Spiegel Online*, only the EU legislature can realistically address this challenge within a meaningful timeframe to avoid impacts on a generation of creators who rely on reuse of copyright and related rights material and uncertainty for all creators, as some level of borrowing underpins all creation. Or as J. Peter Burkholder, one of the foremost scholars in the field of musical borrowing in recent decades, summed up: “If we examined all music borrowed in some way from its predecessors, we would be examining all music”.^182^

## Footnotes

1 While reuse is typically deliberate, we are aware that unintentional, subconscious and accidental reuse also occurs. We focus on examples of deliberate reuse here; as we will explain, unintentional acts are unlikely to satisfy the conditions for lawful quotation, such as attribution.

2 ECJ, 29 July 2019, case C-476-17 – *Pelham v. Hütter*. This decision’s precedents have since been applied by the German Federal Court of Justice, 30 April 2020, case I ZR 115/16 – *Pelham v. Hütter*. ECJ, 29 July 2019, case C-516/17 – *Spiegel Online GmbH v. Volker Beck*; and ECJ, 29 July 2019, case C-469/17 – *Funke Medien v. Bundesrepublik Deutschland*.

3 Information Society Directive, 2001/29/EC.

4 *See* Dutch Copyright Act 2018, Art. 13; German Copyright Act 1965, Sec. 24(1); Norwegian Copyright Act 2018, Sec. 6; and Swedish Copyright Act (1960:729), Sec. 4. Because of COVID-19 limitations on access to research materials, we have not analysed Austria, another state with free use and quotation provisions.

5 How ECJ case law affects interpretation in an EEA context differs depending on whether substantive, procedural or effects-based homogeneity applies. *See* Speitler (2017), pp. 27–28. We can illustrate this in the context of the InfoSoc quotation provision. Under substantive homogeneity, the EFTA Court should adopt any ECJ conditions on quotation under InfoSoc. Under procedural homogeneity, the EFTA Court is not bound to follow ECJ reasoning, but the ECJ reasoning is relevant when the legislative text is identical, which is the case as the EEA has also adopted the InfoSoc Directive. Under effects-based homogeneity, there is some leeway for interpretation by the national EEA courts, though they are obliged to ensure that interpretation of the national law reaches a similar result to interpretation of the EEA law provision.

6 Information Society Directive, 2001/29/EC, Art. 5; ECJ, 29 July 2019, case C-476/17 – *Pelham v. Hütter*, para. 65.

7 For positions in favour of such a continuation, *see e.g*. Haberstumpf (2020a, b); Schulze (2019, 2020). Our position in this regard is summarised in Sect. 4.1, based on a detailed analysis in Döhl (2020b), pp. 242–252.

8 For positions in favour of such a broad understanding of pastiche, *see e.g.* Dreier (2019), p. 1005; Dreier (2020), p. 223; Jütte and Maier (2017), pp. 790–791; Stieper (2020a, b), p. 754. Our position in this regard is explained in Sect. 4.2.

9 For positions in favour of such a broad understanding of pastiche, *see e.g*. Aplin and Bently (2020), p. 124; Hudson (2017), p. 346; Lauber-Rönsberg (2020), p. 738 *et seq.*; Leistner (2020), p. 511; European Copyright Society (2020), p. 125 *et seq*.; Ohly (2020), p. 852; Pötzlberger (2018), pp. 248–282; Rehbinder and Peukert (2018); Senftleben (2019), p. 374 (2020a, b), p. 157 *et seq*.; Stieper (2020a, b), pp. 708, 702 *et seq*.; Stieper (2015), p. 305; Stieper (2020a, b), p. 793 *et seq*., 796 *et seq*.; Wandtke and Hauck (2020), p. 677; Würtenberger and Freischem (2020), p. 1064. Our position in this regard is summarised in Sect. 4.3, based on a detailed analysis in Döhl (2020b). *See* Charter of Fundamental Rights of the European Union, 2000/C 364/01, Arts. 11, 13 and 17.

10 For a position in favour of such a broad understanding of quotation, *see e.g*. Bently and Aplin (2019); Aplin and Bently (2020). Our position in this regard is explained in Sect. 4.4.

11 For a detailed discussion of the position that the free use provisions cannot continue after *Pelham* as has been suggested by some commentators, *see* Döhl (2020c), pp. 242–251.

12 For a detailed discussion of a recent German, post-*Pelham* case in the field of appropriation of art concerning the well-known visual artist Martin Eder that exemplifies this urgency, *see* Döhl (2020b), p. 259 *et seq*. *See also* Berlin Court of Appeals, 30 October 2019, case 24 U 66/19.

13 As a case in point with regard to Germany, *see* Döhl (2020c), p. 259 *et seq*.

14 Information Society Directive, 2001/29/EC.

15 Copyright in the Digital Single Market Directive, (EU) 2019/790.

16 As the Court’s English translation of its decision explains, “[t]here is no entitlement to a licence to use the sample; phonogram producers can refuse a licence on the basis of their right of disposal, without having to give reasons and irrespective of any readiness to pay a fee for the licence”. German Federal Constitutional Court, 31 May 2016, case 1 BvR 1585/13 – *Pelham v. Hütter*, para. 98.

17 This intellectual property concept requires that some common elements remain available for others to use in certain public interest cases. The ECJ has considered this concept and whether the three-stripe adidas trademark prevented other clothing retailers from using two vertical parallel stripes contrasting with the colour of the garment. *See* ECJ, 29 May 2008, case C-102/07 – *adidas v. Marca Mode*.

18 Charter of Fundamental Rights of the European Union, 2000/C 364/01, Arts. 11, 13 and 17.

19 In *Pelham*, the ECJ explicitly declares free use null and void in the context of phonograms if they exceed limitations and exceptions in InfoSoc Art. 5(2) and (3), *see* paras. 56–65. To us, para. 60 indicates that the decision rolls back free use in relation to all rights by referring to InfoSoc Art. 2, not just the phonogram right in Art. 2(c). *See infra* note 41.

20 *See* question 3 referred to the ECJ, and the ECJ’s discussion. ECJ, 29 July 2019, case C-476/17 – *Pelham v. Hütter*, paras. 56–65.

21 ECJ, 29 July 2019, case C-476/17 – *Pelham v. Hütter*, paras. 56–74.

22 *See* German Federal Court of Justice, 30 April 2020, case I ZR 115/16 – *Pelham v. Hütter*, para. 91.

23 Pastiche was not discussed in the Berlin Court of Appeals. Berlin Court of Appeals, 30 October 2019, case 24 U 66/19. *See* in detail Döhl (2020c), p. 259 *et seq*. *See* first instance decision: Berlin District Court, 23 April 2019, case 15 O 102/19.

24 *See* Döhl (2020a, b); Leistner (2019); Ohly (2017); Ohly (2020); Stieper (2020a, b). The main opposing positions still defending continuation of Sec. 24(1) after *Pelham v. Hütter* – *see e.g*. Haberstumpf (2020a, b); Schulze (2019, 2020) – have lost their basis after the decision of the government to repeal Sec. 24(1).

25 *See* Döhl (2020a), German Federal Ministry of Justice’s proposal for an amended German Copyright Act, https://www.bmjv.de/SharedDocs/Gesetzgebungsverfahren/Dokumente/RefE_Urheberrecht.pdf?__blob=publicationFile&v=7, pp. 1, 40.

26 *See* Dutch Copyright Act 2018, Art. 15a; German Copyright Act 1965, Sec. 51; Norwegian Copyright Act 2018, Sec. 29; and Swedish Copyright Act (1960:729), Sec. 22.

27 *See* Bently and Aplin (2019), p. 28; Fischer (2018), p. 71; Leistner (2019), p. 1012; Maier (2018), pp. 43, 198; Papastefanou (2019), p. 601; Schunke (2020), p. 455; Stieper (2020a, b), p. 753. For details, *see also* Aplin and Bently (2020). This scenario seems to be likely to us although not uncontested. For example, the German Federal Ministry of Justice highlights and develops the pastiche exception in this regard. *See* German Federal Ministry of Justice proposal for an amended German Copyright Act, https://www.bmjv.de/SharedDocs/Gesetzgebungsverfahren/Dokumente/RefE_Urheberrecht.pdf?__blob=publicationFile&v=7, pp. 95–97. *See* later Sect. 4.3. For a detailed discussion of the severe problems of this legislative approach, however, *see* Döhl (2020a, b).

28 *See* Aplin and Bently (2020). They go as far as to argue that the InfoSoc Directive quotation provision includes conditions that are inconsistent with the Berne quotation provision, which they consider to require all Berne states to implement a quotation right that is “very close to being a ‘fair use’ exception”, Bently and Aplin (2019), p. 28. Parkin contests Bently and Aplin’s claim, arguing that quotation under UK and EU law is much narrower than US fair use, *see* Parkin (2019).

29 Senftleben (2020b).

30 *See* Aplin and Bently (2020), pp. 226–229, footnote 2.

31 Döhl (2019); Döhl (2020c).

32 Copyright Duration Directive, 2006/116/EC.

33 Döhl (2016).

34 Information Society Directive, 2001/29/EC, Art. 2.

35 At times labelled “*Freihaltebedürfnis*”, *e.g*. in German copyright law. *See e.g*. Wandtke and Bullinger (2019), p. 60.

36 Information Society Directive, 2001/29/EC, Art. 2(c). ECJ, 29 July 2019, case C-476/17 – *Pelham v. Hütter*, para. 31.

37 A free use limitation could be retained in Germany to the extent it codifies InfoSoc exceptions and limitations. For example, in the *Bettina Z* case, the German Federal Court of Justice drew parallels between German free use and the InfoSoc parody exception in cases of parody. German Federal Court of Justice, 28 July 2016, case I ZR 9/15 – *Bettina Z*, para. 27; *see also* Rognstad (2018), p. 105. However, the German Federal Court of Justice has decided in its final *Pelham* judgment that for cases from 22 December 2002 onwards the free use limitation only applies in relation to use that would be consistent with InfoSoc exceptions and limitations implemented by the national legislature (*e.g.* excluding pastiche in the case of Germany). German Federal Court of Justice, 30 April 2020, case I ZR 115/16 – *Pelham v. Hütter*, paras. 38–48, 64 *et seq*. To us, the ECJ is clear enough that this opinion will not be limited to cases of Art. 2(c) InfoSoc Directive. ECJ, 29 July 2019, case C-476/17 – *Pelham v. Hütter*, para 60. This is also the reading of the German Federal Court of Justice, 30 April 2020, case I ZR 115/16 – *Pelham v. Hütter*, para. 91. *See also* the “German section” below for further arguments.

38 The German Federal Court of Justice has opted for a human lay-listener test in its latest *Pelham* judgment, continuing its former position in this regard. *See* Döhl (2015); and German Federal Court of Justice, 30 April 2020, case I ZR 115/16 – *Pelham v. Hütter*.

39 US Ninth Circuit Court of Appeals, (as amended) 11 July 2018 – *Williams v. Gaye*.

40 Information Society Directive, 2001/29/EC, Art. 5(3)(k).

41 ECJ, 3 September 2014, case C-201/13 – *Deckmyn v. Vandersteen*.

42 ECJ, 3 September 2014, case C-201/13 – *Deckmyn v. Vandersteen*. *See* Döhl (2016) for a detailed discussion of such difficulties in instrumental music.

43 *See* Bently et al. (2019), p. 470. Information Society Directive, 2001/29/EC, recital 31.

44 *See* for a detailed discussion of all arguments, Döhl (2020b).

45 *See* Döhl (2019) regarding the legal interpretation of pastiche, *e.g*. in France and Belgium, or the interpretation of “similar transformations” (*vergleichbare Abwandlungen*) in Switzerland; *see* Döhl (2020a) for a newly proposed, very broad German reading of pastiche that is in stark contrast to the rather limited French regulation.

46 *See* Döhl (2019, 2020a).

47 German Copyright Act 1965, Sec. 24(1). There have been many German studies on this concept during the last quarter of a century, *e.g*. Fischer (2018); Wegmann (2013); Wehler (2012). Dutch Copyright Act 2018, Art. 13. Regarding the German provision, *see also* Döhl (2020a, b, c).

48 Norwegian Copyright Act 2018, Sec. 6. Swedish Copyright Act 1960:729, Sec. 4.

49 *See* for the introduction of the argument, German Federal Court of Justice, 5 November 1957, case I ZR 83/56 – *Sherlock Holmes*; German Federal Court of Justice, 4 February 1958, case I ZR 48/57 – *Lili Marleen*; German Federal Court of Justice, 1 April 1958, case I ZR 49/57 – *Mecki-Igel*.

50 Norwegian Supreme Court, 15 November 2017, HR-2017-2165-A – *Aukruststiftelsen v. Caprino Filmcenter*. The discussion here is based on the Supreme Court of Norway’s English translation, noting the authoritative text is in Norwegian.

51 In this case, the rollercoaster carriage possessed “an industrial look and gives a completely different aesthetic impression than the film car’s refined artistic look”; thus the rollercoaster carriage did not infringe on the adaptation copyright in the car which was limited by being based on the drawings. *Ibid*., paras. 71, 73, 85, 117.

52 *See* Döhl (2015).

53 Dutch Supreme Court, 28 June 1946, NJ 1946 – *Van Gelder v. Van Rijn.* In the later *Barbie* decision, the Dutch Supreme Court established an evidentiary presumption rule; if the allegedly infringing work has a strong resemblance to a prior copyright work, courts may assume copying unless the defendant proves otherwise. Dutch Supreme Court, 21 February 1992, NJ 1993 – *Snijders, Hermans, Bloembergen, Haak, Heemskerk v. A-G Asser* (*Barbie/Sindy*).

54 Dutch Supreme Court, 29 November 2002, NJ 2003 – *Eiser v. Verweersters*.

55 *See* Swedish Supreme Court, NJA 1975 p. 679 – *Sveriges Flagga*; Swedish Supreme Court, 23 December 2005, NJA 2005 p. 905 – *GB v. Sveriges Radio*; Swedish Supreme Court, 23 February 2017, NJA 2017 p. 75 – *JL v. MA*.

56 German Federal Court of Justice, 28 July 2016, case I ZR 9/15 – *Bettina Z*, para. 27.

57 Swedish Supreme Court, 23 February 2017, NJA 2017 p. 75 – *JL v. MA*. *Parodier* and *travestier* in Swedish translates approximately to parodies, caricatures, burlesques and travesties. Swedish Government Copyright in literary and artistic works, Right to photographic images Bill 1960, NJA II 1961, p. 81. *See* Jacques’ discussion of the related meanings of travesty, parody, burlesque and caricature. Jacques (2019), p. 11.

58 The painting was also exhibited in a museum and copies were later sold as posters.

59 Swedish Supreme Court, 23 February 2017, NJA 2017 p. 75 – *JL v. MA*, para. 14.

60 Swedish Supreme Court, 23 December 2005, NJA 2005 p. 905 – *GB v. Sveriges Radio*, Sec. 3.

61 Swedish Supreme Court, 23 December 2005, NJA 2005 p. 905 – *GB v. Sveriges Radio*. *See also* Olsson (1996), p. 86.

62 German Copyright Act 1965, Sec. 24(2).

63 Information Society Directive, 2001/29/EC, Art. 5(3)(d).

64 Berne Convention for the Protection of Literary and Artistic Works, as amended on 28 September 1979, Art. 10(1).

65 WIPO Copyright Treaty, Art. 1, requires compliance with Berne Convention, Arts. 1 to 21. Art. 10(1) of the Berne Convention states “it shall be permissible” to make certain quotations. *See* Rosati (2017), pp. 313–314; and Information Society Directive, 2001/29/EC, recital 15. Liechtenstein is also a signatory to the WIPO Copyright Treaty, although other non-EU EEA states (Norway, Iceland) are not.

66 Dutch Supreme Court, 3 April 2015, 14/01158, 5.1.4. – *GS Media v. Sanoma Media Netherlands*.

67 For example, the Swedish Writers’ Union refers to “*citaträtten*”. Sveriges Författarförbund (c2019), *Hur mycket får man citera ur en text?*, available at: https://forfattarforbundet.se/radgivning/fragorochsvar/hur-mycket-far-man-citera-ur-ett-verk/. *See also* the Svenska Förläggareföreningen (the Swedish Publishers’ Association, c2019), *Upphovsrätt*, available at: https://www.forlaggare.se/upphovsratt. Norwegian examples include Den norske forleggerforening, Den norske historiske forening og Norsk faglitterær forfatter- og oversetterforening [Norwegian Publishers Association, Norwegian Historical Association, Norwegian Non-fiction Writer and Translator Association] (2006), *God skikk: Om bruk av litteratur og kilder i allmenne, historiske framstillinger*, available at: https://forleggerforeningen.no/wp-content/uploads/2019/03/God-skikk-Om-bruk-av-litteratur-og-kilder-i-allmenne-historiske-framstillinger.pdf.

68 Dutch Copyright Act 2018, Art. 15a. For this section, we rely on Mireille van Eechoud’s translation, *see* van Eechoud (2012). *See also* Senftleben (2012). The Dutch Supreme Court has recognised the quotation provision as a right, referring to it as “*citaatrecht*”. Dutch Supreme Court, 3 April 2015, 14/01158, 5.1.4. – *GS Media v. Sanoma Media Netherlands*.

69 For the general discourse about quotation and its legal requirements, *see* Ahlberg and Götting (2019), Sec. 51, paras. 1–26; Dreier et al. (2018), Sec. 51, paras. 1–20; Wandtke and Bullinger (2019), Sec. 51, paras. 1–20. *See* German Copyright Act, Secs. 51, 62 and 63.

70 Swedish Copyright Act 1960:729, Sec. 22. Norwegian Copyright Act 2018, Sec. 29. The Swedish *citat*, Norwegian *sitat*, Dutch *citaat* and German *Zitat* all translate to both quotation and citation. There is little superior court precedent on the Swedish provision.

71 The Norwegian provision limits quotation “to the extent and in the form required by the purpose”; similarly, the Swedish provision limits it “to the extent justified by the purpose”; and the German provision requires the quotation is “justified to that extent by the particular purpose”. Norwegian Copyright Act 2018, Sec. 25. Swedish Copyright Act 1960:729, Sec. 22. German Copyright Act, Sec. 51.

72 Dutch Copyright Act 2018, Art. 25.

73 The film *Brent av Frost* (or Burnt by Frost) featured a sex scene involving Mauseth. A later parody of *Brent av Frost* featured an excerpt from this sex scene, including two seconds which showed Mauseth nude. *See* Norwegian Supreme Court, 15 November 2017, HR-2017-2165-A – *Aukruststiftelsen v. Caprino Filmcenter*.

74 The freedom of speech has been recognised in Norwegian law since the 1814 Constitution. It is currently codified in the Norwegian Basic Law 2014, Sec. 100. The Supreme Court recognised that actor Mauseth’s claim was related to freedom of speech: “*Mauseths krav er prinsipielt sett et inngrep i ytringsfriheten*”.

75 Swedish Copyright Act 1960:729, Sec. 22; Norwegian Copyright Act 2018, Sec. 29; Dutch Copyright Act 2018, Art. 15a(1)(2).

76 *Tweede Kamer 2001–2002*, 28 482, Nr. 3 *(Memorie van Toelichting*), p. 40.

77 German Copyright Act 1965, Sec. 63; Norwegian Copyright Act 2018, Sec. 29; Swedish Copyright Act 1960:729, Sec. 22.

78 Dutch Copyright Act 2018, Sec.15a(1)(1).

79 German Copyright Act 1965, Sec. 63.

80 *Ibid*.

81 Norwegian Copyright Act 2018, Sec 5.

82 Dutch Copyright Act 2018, Sec. 15a(1)(4).

83 Swedish Copyright Act 1960:729, Secs. 3, 11, 22.

84 Norwegian Copyright Act 2018, Sec. 5; Swedish Copyright Act 1960:729, Secs. 3, 11.

85 Dutch Copyright Act 2018, Sec. 25.

86 Alteration or lack of attribution does not necessarily lead to an act of unlawful quotation but it generates the possibility of claims for cease and desist orders as well as claims for damages. German Copyright Act, Sec. 62. Alterations are permitted for uses by a rightholder other than the author, where the author cannot deny consent in good faith. German Copyright Act, Sec. 39.

87 *See* Nordemann et al. (2018), § 62, marginal note 15, and § 39, marginal note 9.

88 *See* Ahlberg and Götting (2019), Sec. 51, paras. 1–26; Dreier et al. (2018), Sec. 51, paras. 1–20; Wandtke and Bullinger (2019), Sec. 51, paras. 1–20. *See* German Copyright Act, Secs. 51, 62 and 63.

89 German Copyright Act, Sec. 51. *See* Wandtke and Bullinger (2019), Sec. 51, para. 20.

90 *Tweede Kamer 2001–2002*, 28 482, Nr. 3 *(Memorie van Toelichting*), p. 40.

91 Information Society Directive, 2001/29/EC, Art. 5(3)(d).

92 Information Society Directive, 2001/29/EC, Art. 5(3)(d).

93 Information Society Directive, 2001/29/EC, Art. 5(3)(d).

94 This is more in line with Berne, which also specifies no purpose. Berne Convention for the Protection of Literary and Artistic Works, as amended on 28 September 1979, Art. 10(1).

95 Dutch Copyright Act 2018, Art. 15a.

96 Dutch Court of Appeals of Arnhem, 4 July 2006, AMI 2007, p. 93.

97 Many of the points we make here will also be relevant to other EU and EEA states without an InfoSoc-style quotation exception. For example, Spain has a quotation exception that is limited to education or scientific purposes, which is materially different from the criticism or review purposes permitted by InfoSoc.

98 ECJ, 29 July 2019, case C-476/17 – *Pelham v. Hütter*, para. 71.

99 *See e.g*. German Constitutional Court, 29 June 2000, case 1 BvR 825/98 – *Germania 3*, para. 26.

100 In the context of the German free use provision, there were further complications. The German Federal Court of Justice, for example, disagreed partly and requested an “inner connection” between the source and the thoughts and ideas of the reuser; in the context of music, this would have permitted quotations for the purpose of creating contrast or for purely stylistic means but only if the quoted material was recognisable as being quoted material. Overall, it becomes clear that there are severe, unresolved conflicts for quotation in the arts when it comes to the requirement of purpose – which goes along with significant legal uncertainty for the reuser, a much more complicated scenario to evaluate than estimating if substantial distancing is achieved as it is solely requested in the context of free use. *See e.g*. German Federal Court of Justice, 20 December 2007, case I ZR 42/05 – *TV Total*, para 42; German Federal Court of Justice, 30 April 2020, case I ZR 115/16 – *Metall auf Metall IV*, para. 53.

101 ECJ, 29 July 2019, case C-476/17 – *Pelham v. Hütter*, paras. 71–72. *See also* Advocate General Szpunar’s opinion: “Exceptions to the exclusive rights such as the quotation, and caricature, parody and pastiche exceptions facilitate dialogue and artistic confrontation through references to pre-existing works”. Opinion of Advocate General, ECJ, case C-476/17 – *Pelham v Hütter*, para. 95.

102 ECJ, 29 July 2019, case C-476/17— *Pelham v. Hütter*, paras. 71–72.

103 ECJ, 29 July 2019, case C-476/17— *Pelham v. Hütter*, para. 73.

104 In the respective free use provision, if the source is not recognisable, it is a case of free use. If it is identifiable, then it becomes a matter of sufficient distancing. The burden of proof relating to “not possible to identify the work” lies with the user, but proving “unrecognisability of the source” falls upon the rightholder.

105 Information Society Directive, 2001/29/EC, Art. 5(3)(d).

106 The test permits Berne member countries to permit reproduction of certain works that satisfy three steps: the permission is granted in (1) certain special cases, provided that (2) such reproduction does not conflict with a normal exploitation of the work and (3) does not unreasonably prejudice the legitimate interests of the author. Berne Convention for the Protection of Literary and Artistic Works, as amended on 28 September 1979, Art. 9(2).

107 ECJ, 29 July 2019, case C-516/17 – *Spiegel Online v. Volker Beck*, para. 79.

108 Berne Convention for the Protection of Literary and Artistic Works, Paris Revision (24 July 1971, entered into force 10 October 1974), Art. 10(1).

109 Information Society Directive, 2001/29/EC, Art. 5(3)(d).

110 These provisions are closer to the Berne quotation requirement that quoted works merely be published. Berne Convention for the Protection of Literary and Artistic Works, Paris Revision (24 July 1971, entered into force 10 October 1974), Art. 10(1).

111 *See* Ohly (2017), p. 967.

112 *See* ECJ, 29 July 2019, case C-476/17 – *Pelham v. Hütter*, paras. 77, 81.

113 *See* Rosati (2020), p. 267.

114 *See* ECJ, 16 July 2009, case C-5/08 – *Infopaq*, paras. 39–42.

115 *See* German Federal Court of Justice, 16 May 2013, case I ZR 28/12 – *Beuys-Aktion*, tenor 1, para. 37. *See also* German Federal Court of Justice, 30 April 2020, case I ZR 115/16 – *Metall auf Metall IV*, para. 36.

116 For example, in *Pelham*, the ECJ opined that the InfoSoc phonogram reproduction right must be interpreted as constituting a measure of full harmonisation. Information Society Directive, 2001/29/EC, Arts. 2, 3, 4. ECJ, 29 July 2019, case C-476/17 – *Pelham v. Hütter*, para. 65. ECJ, 29 July 2019, case C-469/17 – *Funke Medien v. Bundesrepublik Deutschland*, paras. 35–38.

117 ECJ, 29 July 2019, case C-476/17 – *Pelham v. Hütter*, para. 65.

118 ECJ, 29 July 2019, case C-516/17 – *Spiegel Online GmbH v. Volker Beck*, para. 33.

119 The quote in this passage is from the referring court’s question. It appears the intended meaning in this passage is “affect”, not “effect”. ECJ, 29 July 2019, case C-469/17 – *Funke Medien v. Bundesrepublik Deutschland*, para. 33.

120 *See e.g*. Dreier (2020); Geiger and Izyumenko (2019), p. 5; Handig (2019), p. 498; Leistner (2019), p. 1011; Marly and Prinz (2019); Oechsler (2020), p. 3208; Rognstad (2020), pp. 85–86; Schulze (2020), p. 131; Stieper (2019), p. 715 *et seq*.

121 Geiger and Izyumenko (2019), p. 5.

122 *See e.g*. Burkholder (2020).

123 *See* ECJ, 29 July 2019, case C-469/17 – *Funke Medien v. Bundesrepublik Deutschland*, paras. 30–38, 45–53.

124 ECJ, 29 July 2019, case C-476/17 – *Pelham v. Hütter*, para. 64.

125 *See* the reasoning of the German Constitutional Court, 31 May 2016, case 1 BvR 1585/13 – *Pelham v. Hütter*.

126 Jütte (2020), p. 80; Information Society Directive, 2001/29/EC, Art. 5.

127 Engman highlights the EU law considerations of proportionality, high protection for rightholders and regard for the internal market, the three-step test and the balancing of EU fundamental rights. Engman (2020), p. 130.

128 Rosati (2020), p. 267.

129 Charter of Fundamental Rights of the European Union, 2012/C 326/02.

130 *See* Dreier (2019), p. 1005; Dreier (2020), p. 223; Jütte and Maier (2017), pp. 790–791; Stieper (2020a, b), p. 754.

131 *See* ECJ, 29 July 2019, case C-476/17 – *Pelham v. Hütter*, paras. 31, 34.

132 *See* Dreier (2019), p. 1005; European Copyright Society (2020), pp. 126–127; Goldhammer (2019), p. 729.

133 *See* ECJ, 29 July 2019, case C-469/17 – *Funke Medien v. Bundesrepublik Deutschland*, para. 32.

134 Charter of Fundamental Rights of the European Union, 2000/09, Arts. 11, 13.

135 ECJ, 29 July 2019, case C-476/17 – *Pelham v. Hütter*, paras. 56–65.

136 *See* Oechsler (2020), p. 3208.

137 van Deursen and Snijder also note, “it is argued that it follows from the current line of case law that the limiting role of other fundamental rights is restricted to specific enforcement measures aimed at intermediaries and the interpretation of the existing limitations and exceptions and that they cannot fulfil an external balancing role”. Geiger and Izyumenko (2020), pp. 298–302; Quintais et al. (2020); Snijders and van Deursen (2019), p. 1185; van Deursen and Snijders (2020), p. 1088; Senftleben (2020b), pp. 764–765.

138 *See* Jongsma (2020); Jütte and Maier (2017), p. 787; Senftleben (2020b), p. 765.

139 *See* Bently et al. (2018), p. 475.

140 *See* ECJ, 29 July 2019, case C-469/17 – *Funke Medien v. Bundesrepublik Deutschland*, paras. 37, 51.

141 *See* Bently et al. (2018), p. 474.

142 *See* Quintais et al. (2020), p. 278, para. 11; Rosati (2020), p. 266; Stieper (2019), p. 715; The Bird & Bird IP Team (2020), p. 341.

143 *See* Jütte (2019), p. 828.

144 ECJ, 29 July 2019, case C-476/17 – *Pelham v. Hütter*, paras. 32–34, 59–61.

145 ECJ, 3 September 2014, case C-201/13 – *Deckmyn v. Vandersteen*.

146 We expect non-EU EEA states will also be obligated to implement the DSM Directive once it is adopted into EU law.

147 Copyright in the Digital Single Market Directive, (EU) 2019/790, Art. 17(7)(a) and recital 70.

148 *See* German Federal Ministry of Justice’s proposal for an amended German Copyright Act, https://www.bmjv.de/SharedDocs/Gesetzgebungsverfahren/Dokumente/RefE_Urheberrecht.pdf?__blob=publicationFile&v=7, p. 97. This German reaction towards the *Pelham* judgment is extensively discussed in Döhl (2020c).

149 *See* Ohly (2017), p. 967.

150 *See* Döhl (2020c), pp. 251–260.

151 Information Society Directive, 2001/29/EC, Art. 5(3)(k). *See* Aplin and Bently (2020), p. 124; Bently et al. (2019), p. 374; European Copyright Society (2020), p. 125 *et seq*.; Hudson (2017), p. 346; Lauber-Rönsberg (2020), p. 738 *et seq*.; Leistner (2020), p. 511; Ohly (2020), p. 852; Pötzlberger (2018), pp. 248–282; Rehbinder and Peukert (2018), p. 182; Senftleben (2020a), p. 157 *et seq*.; Stieper (2015), p. 305; Stieper (2020a), p. 702 *et seq*.; Stieper (2020b), pp. 793 *et seq*., 796 *et seq*.; Wandtke and Hauck (2020), p. 677; Würtenberger and Freischem (2020), p. 1064.

152 For a detailed explanation, *see* Döhl (2020b).

153 Döhl (2020c), pp. 251–260.

154 *See* Döhl (2020b), pp. 404–442.

155 *See* Aplin and Bently (2020), p. 124.

156 *See also* Bently et al. (2018), p. 252; Döhl (2020a), p. 745; Döhl (2020b), p. 439; Gelke (2013), p. 177; Griffiths (2017), p. 84; Haberstumpf (2020b), p. 106; Haberstumpf (2020a), p. 814; Jacobsen (2020), p. 10; Jacques (2015), p. 702; Jacques (2019), p. 11; Jütte (2014), p. 182; Lambrecht and Cabay (2015), p. 371; Lerach (2020); Maier (2018), p. 61; Marshall (2018), p. 958; Schack (2019), p. 153; Schulze (2020), p. 129; Vlah (2015), p. 50, para. V. Despite the contrary views of advocates of a broad pastiche concept, such an understanding of pastiche has been the basis of successful claims in music, for example in the US *Blurred Lines* or German *Sadeness (Part I)* judgments. *See* Döhl (2020b), p. 624 *et seq*.

157 For an analysis of the scope of these challenges, *see* Döhl (2020c), p. 255 *et seq*.

158 For detailed lists of the dozens of legal comments on *Pelham* and its German predecessors that did not take the pastiche exception into account, *see* Döhl (2016), p. 208, note 15 (comments until 2008); Döhl (2018), pp. 280–281, note 39 (between 2008 and 2016); and Döhl (2020b), pp. 388–389, notes 36–37 (all comments regarding the ECJ stage). Only some comments mention the possibility that the pastiche exception might apply but do not argue in favour of it, *see* Apel (2018), p. 190; Brtka (2020), p. 586; Czychowksi and Düstersiek (2020), pp. 213–216; Grünberger (2020), p. 201; Lerach (2020), para. 8; Ohly (2017), pp. 968–969; Ohly (2020), p. 852; Pötzlberger (2019), p. 252; Schulze (2020), p. 129; Stumpf (2019), p. 1095.

159 Aplin and Bently (2020), pp. 138–139; Jütte and Maier (2017), p. 794; Senftleben (2020b), pp. 764–765.

160 Bently and Aplin (2019), p. 8.

161 Berne Convention for the Protection of Literary and Artistic Works, Paris Revision (24 July 1971, entered into force 10 October 1974), Art. 9.

162 *Ibid*., Arts. 9, 10(1), 10^bis^.

163 Aplin and Bently (2020), pp. 226–229.

164 *See e.g*. European Copyright Society (2020), p. 126, para. 38, referring to the ECJ’s *Painer* decision.

165 *See e.g*. German Constitutional Court, 29 June 2000, case 1 BvR 825/98 – *Germania*, para. 26.

166 *See* Aplin and Bently (2020), pp. 226–229.

167 *See* Parkin (2019), p. 87.

168 We raise a similar objection to a similarly broad understanding of pastiche in Sect. 4.3.

169 Not going in the direction of a broadening of the InfoSoc quotation provision are, for example, European Copyright Society (2020); Quintais et al. (2020); Snijders and van Deursen (2019).

170 *See* Haberstumpf (2020a), p. 812

171 The same is, for example, true for the limited “purposes such as criticism or review” listed in InfoSoc which does not suit reuse in music well.

172 *See e.g*. Burkholder (1994).

173 This is a version of the Berne three-step test, though we emphasise its reappearance in EU law. *See* Rosati (2020), p. 266.

174 Rosati (2020), p. 267.

175 Copyright in the Digital Single Market Directive, (EU) 2019/790.

176 *See*, *e.g.*, Sec. 51a of a German Federal Ministry of Justice proposal for an amended German Copyright Act, https://www.bmjv.de/SharedDocs/Gesetzgebungsverfahren/Dokumente/RefE_Urheberrecht.pdf?__blob=publicationFile&v=7. Copyright in the Digital Single Market Directive, (EU) 2019/790, Art. 17(7). Art. 17(7) stands alongside Art. 17(1) requirements for online content-sharing service providers to obtain authorisation (such as a licence) for copyright material on their service, and to ensure unavailability of unauthorised or infringing material on their service.

177 This was before digital appropriation and distribution of arts via the world wide web, including mp3, P2P systems (Napster etc.), video platforms (YouTube etc.), social media (Facebook, Twitter, Instagram etc.), and streaming services (Spotify, SoundCloud etc.).

178 *See* German Federal Constitutional Court, 31 May 2016, case 1 BvR 1585/13 – *Pelham v. Hütter*, para. 86.

179 ECJ, 3 September 2014, case C-201/13 – *Deckmyn v. Vandersteen*.

180 *See* Döhl (2020b), p. 386, for an extensive list of demands and proposals in this regard in the legal literature of the last 15 years.

181 For a detailed discussion of these consequences, *see* Döhl (2020c).

182 Burkholder (1994), p. 863.
